# Comparative Analysis of Oxygen Saturation by Pulse Oximetry and Arterial Blood Gas in Hypoxemic Patients in a Tertiary Care Hospital

**DOI:** 10.7759/cureus.42447

**Published:** 2023-07-25

**Authors:** Elen A Abraham, Ghanshyam Verma, Yasar Arafat, Sourya Acharya, Sunil Kumar, Nikhil Pantbalekundri

**Affiliations:** 1 Department of Respiratory Medicine, Sree Balaji Medical College and Hospital, Chennai, IND; 2 Department of Medicine, Jawaharlal Nehru Medical College, Datta Meghe Institute of Medical Sciences (Deemed to be University), Wardha, IND; 3 Department of Medicine, Jawaharlal Nehru Medical College, Wardha, IND; 4 Department of Medicine, Jawaharlal Nehru Medical College, Sawangi Meghe, Wardha, Wardha, IND

**Keywords:** respiratory disease, arterial blood gas, pulse oximetry, oxygenation, acute hypoxemia

## Abstract

Introduction: Oxygen saturation is essential for medical care and is closely regulated within the body. Arterial blood gas (ABG) analysis is used to evaluate critically ill individuals' ventilation, oxygenation, acid-base status, and metabolic condition. Pulse oximetry is an easy and non-invasive way to measure the status of oxygen saturation non-invasively in clinical settings and provides a quick and precise assessment of oxygenation and reduces medical errors. SpO_2_ may not always be a reliable predictor of arterial oxygen saturation (SaO_2_), and hypoxemic, hemodynamically compromised, and critically ill patients may have lower SpO_2_ accuracy. A study is needed to assess and compare various oxygen saturation methods.

Aims and objectives: The study aimed to compare the oxygen saturation levels measured by pulse oximetry and ABG analysis in hypoxemic patients. The objectives were to compare the values between SaO_2_, PaO_2_, and SpO_2_ values obtained from the patients, and correlate the study parameters among both techniques.

Materials and methods: The study was conducted from February 2021 to June 2022 among the 102 hypoxemic patients who were admitted to the emergency and surgical intensive care unit (ICU) of Sree Balaji Medical College and Hospital in Chennai. Primary data on ABG analysis and pulse oximetry readings were collected from the study subjects. The patient and their past medical records, physical exam, chest x-ray findings, pulse oximetry, and ABG results were all reviewed. Each patient had their ABG, and pulse oximetry measured simultaneously. A comparison was made between SpO_2_ and partial pressure of oxygen (PaO_2_) and arterial oxygen saturation (SaO_2_) parameters using a paired t-test. The correlation was done against the SpO_2_ and ABG parameters and assessed for association using the correlation coefficient value; gender was also considered while correlating.

Results and discussion: An observational study was done among 102 study samples to comparatively analyze the oxygen saturation by two methods, namely pulse oximetry and ABG, in hypoxemic patients. While comparing the mean values of SaO_2_ and SpO_2_, they were 84.41 ± 4.24 and 80.58 ± 5.77, respectively, and this difference was statistically very significant (p < 0.001). While comparing the mean values of PaO_2_ and SaO_2_, they were 61.02 ± 5.01 and 84.41 ± 4.24, respectively, and this difference was statistically significant (p = 0.043). While comparing the mean values of PaO_2_ and SpO_2_, they were 61.02 ± 5.01 and 80.58 ± 5.77, respectively, and this difference was statistically significant (p = 0.054). Among the study population, with regard to the correlation factor, there is notably a very high and strong positive correlation between SaO_2_ and SpO_2_ and between SpO_2_ and PaO_2_. There was a negative correlation between SpO_2_ and finger abnormalities and between SpO_2_ and blood pressure.

Conclusion: The ABG method is considered the gold standard. When SpO_2_ levels fall below 90%, pulse oximetry may not be accurate enough to reliably assess oxygenation. In such cases, where alveolar hypoventilation is suspected, it is recommended to complement pulse oximetry with ABG studies. This is because ABG analysis provides a more comprehensive assessment of oxygenation and acid-base status, which can aid in the diagnosis and management of respiratory conditions.

## Introduction

Analysis of arterial blood gas (ABG) is frequently used to evaluate the ventilation, oxygenation, and acid-base status of critically unwell individuals. ABGs are obtained from arteries and provide a real-time indicator of the patient's oxygenation, ventilation, acid-base balance, and metabolic condition. Samples can be arterial, venous, or a combination of both. An arterial line blood sample or an arterial puncture can be used to measure an ABG. In critically ill patients, ABG analysis is crucial for making the correct diagnosis and selecting the best course of treatment. Respiratory failure is a good example of this function. ABG is the only study capable of identifying, describing, and quantifying respiratory failure. Primary oxygenation failure is indicated by the low or normal partial pressure of arterial carbon dioxide (PaCO_2_), low partial pressure of arterial oxygen (PaO_2_), and an alkaline pH, whereas ventilatory failure is indicated by the highest PaCO_2_, low PaO_2_, and pH. Pulse oximetry is used to measure the status of oxygen saturation (SpO_2_) non-invasively. It is a technique that is frequently utilized in clinical settings, including intensive care, surgery, and even outpatient clinics [1]. Pulse oximetry has been used in these contexts to lessen the ABG analysis and to improve the identification of conditions like hypoxic factors, which is indicated by an observed SpO_2_ value of less than 95% [2].

The rationale of the study was to analyze and compare finger oximeter and ABG readings in hypoxemic patients at a tertiary care hospital in Chennai. Technical issues with the measurement instrument or poor data interpretation can impact the precision and accuracy of SpO_2_ values. Pulse oximetry is an easy, non-invasive way to detect continuous blood oxygen saturation. Previous research has suggested that SpO_2_ may not always be a reliable predictor of SaO_2_. A study must be designed to assess and compare various oxygen saturation methods to determine the most reliable and effective method.

## Materials and methods

The study was carried out among the 18-80 years old hypoxemic population who visited and got admitted to Sree Balaji Medical College and Hospital in Chennai for a period of 17 months (February 2021 to June 2022). The nature and purpose of the study were explained to the Institutional Review Board, Sree Balaji Medical College, and the Hospital following which ethical clearance was obtained to conduct the study (Institutional Human Ethical Committee of Sree Balaji Medical College and Hospital Ref No: 002/SBMC/IHEC/2021/1523). From February 2021 to June 2022, 102 patients at the emergency and surgical ICU at the Sree Balaji Medical College and Hospital in Chennai were the subject of the study. The purpose of the study was to compare and confirm the variables influencing the accuracy of the pulse oximeter in relation to ABG.

Primary data on ABG analysis and pulse oximetry readings were collected from the study subjects. The patient and their past medical records, physical exam, chest x-ray findings, pulse oximetry, and ABG results were all reviewed. After obtaining informed consent, patients who were admitted to the ICU throughout the study period, whether or not they had hypoxemia, were included in the study. Each patient admitted to the ICU had their ABG and pulse oximetry measured simultaneously. Before performing the Allen test, which evaluates the blood supply in the hands, the patient was asked to clench their fist for 30 seconds. After that, the pressure was put over the ulnar and radial arteries, and the hand was then opened. The Monitor - Nellcor Pulse Oximetry DS-100A Adult Finger Sensor was used to measure and analyze the pulse oximetry when the ABG was stopped in compliance with COBAS B 221 standards. A pulse oximeter was used to monitor SpO_2_ (Fingertip, China). In the arm opposite the one from which the arterial sample had been taken the finger probe device was placed on the index finger. SpO_2_ was measured twice at 0 and 2 minutes, and the average of the two measurements was calculated. In this ABG values served as a reference. ABG defines hypoxemia as having a partial oxygen pressure (PaO_2_) below 60 mm Hg or having a SpO_2_ or SaO_2_ below 90% [3].

The subjects included in the study were subjects consenting to the study, a population of 18-80 years of hypoxemic patients, pulse oximetry, and ABG analysis should be performed. simultaneously. The exclusion factors were individuals under the age of 18, patients with fingernails colored, blood samples out of a vein, and patients with methemoglobinemia and carbon monoxide poisoning were also excluded.

The sample size was determined from the G power statistical software tool. Values are recorded and noted in an Excel sheet decoded and coded against the standard values. Descriptive data and inferential statistics were calculated by Paired t-test and Pearson rank correlation coefficient test. Comparison made between SpO_2_ with PaO_2_, SaO_2_ parameters using paired t-test. Correlation was done against the SpO_2_ and ABG parameters and assessed for association using the correlation coefficient value, gender was also considered while correlating.

Statistical analysis version 21 of SPSS was used for the statistical analysis (IBM Corp., Armonk, NY, USA). Excel was used to first tabulate the data. The collected information was quantitative in nature; thus, the normality test was performed on it (Kolmogorov-Smirnov test). Since the data were determined to be regularly distributed, intra- and inter-group comparisons were made using parametric tests of significance. Mean and standard deviation was used to describe descriptive data that was quantitative in nature. SapO_2_ and SaO_2_ were compared using a paired t-test. Using Pearson's correlation, it was determined whether there was a correlation between vital signs and ABG parameters, vital signs and SapO_2_, hemoglobin concentration and ABG parameters, hemoglobin concentration, and SapO_2_, and ABG parameters and SapO_2_. In all tests, a p-value of <0.05 was considered the level of statistical significance.

## Results

An observational study was done among 102 study samples to comparatively analyze the oxygen saturation by two methods namely: (1) Pulse oximetry and (2) ABG in hypoxemic patients in a tertiary care hospital in Chennai. The quantitative data were described using the mean and standard deviation. All continuous variables underwent the Kolmogorov-Smirnov test to evaluate their normality, and the results showed that the data were normally distributed. Therefore, both within-group and between-group comparisons were made using parametric tests of significance.

Table 1 shows the distribution of participants through each stage of the study. A total of 102 adults residing in Chennai, who complained of breathlessness were included in the study. The gender was distributed as 54 males and 48 females (Table 1). Study parameters like PaCO_2_, PaO_2_, and HCO_3_ for ABG and SpO_2_ for pulse oximeter are mentioned.

**Table 1:** Gender distribution of the study samples

Gender	Frequency (n=102)	Percentage (%)
Females	48	47.05
Males	54	52.94

Every patient underwent both kinds of oxygen saturation techniques simultaneously. Values like PaCO_2_, PaO_2_, and HCO_3_ for ABG and SpO_2_ for pulse oximeter were recorded.

Mean age of the study subjects

The mean age of the study population was 49.60 ± 17.09 years.

Mean values of the study parameters

The mean value of hemoglobin content was 9.70 ± 1.24. The mean value of blood pressure was 109.68 + 26.6. The mean value of pH was 7.35 ± 0.10. The mean value of PaO_2_ was 61.02 ± 5.01. The mean value of PaCO_2_ was 38.01 ± 7.54. The mean value of HCO_3_ was 18.51 ± 4.18. The mean value of SaO_2_ was 84.41 ± 4.24. The mean value of SpO_2_ was 80.58 ± 5.77 (Table 2).

**Table 2:** Descriptive statistics depicting the mean values of the study values

Study Parameters	N	Mean	Std. Deviation
Age (in years)	102	49.6078	17.09968
Hemoglobin (g/dL)	102	9.7049	1.24243
BP (mm Hg)	102	109.6863	26.69037
pH	102	7.3597	0.10102
PaO_2 _(mm Hg)	102	61.0294	5.01178
PaCO_2 _(mm Hg)	102	38.0196	7.54784
HCO_3 _(mmol/L)	102	18.5196	4.18798
SaO_2 _(%)	102	84.4118	4.24346
SpO_2 _(%)	102	80.5882	5.77011
Finger abnormality	102	NA	NA

The mean values of each study parameter are listed along with its standard deviation. Since it is a quantitative measurement, the mean and standard deviations are listed in the above-mentioned table. Since finger abnormalities are a qualitative measure, they cannot be quantified.

While comparing the mean values of SaO_2_ and SpO_2_ were 84.41 ± 4.24 and 80.58 ± 5.77, respectively, and this difference was statistically very highly significant (p< 0.001, Table 3). While comparing the mean values of PaO_2_ and SaO_2_ were 61.02 ± 5.01 and 84.41 ± 4.24, respectively, and this difference was statistically significant (p = 0.043, Table 3). While comparing the mean values of PaO_2_ and SpO_2_ were 61.02 ± 5.01 and 80.58 ± 5.77, respectively, and this difference was statistically significant (p = 0.054, Table 3). While comparing the mean values of BP and SpO_2_ were 109.68 ± 26.69 and 80.58 ± 5.77, respectively, and this difference was statistically highly significant (p= 0.007, Table 3). While comparing the mean values of BP and SaO_2_ were 109.68 ± 26.69 and 84.41 ± 4.24, respectively, and this difference was statistically very highly significant (p = 0.001, Table 3).

**Table 3:** Comparison of different variables *Statistically significant; **Statistically highly significant; ***Statistically very highly significant

Comparison of the Gold standard ABG (SaO_2_) and Pulse oximetry readings (SpO_2_)				
		Mean	Standard deviation	P-value
Pair 1	SaO_2_ (%)	84.4118	4.24346	<0.001***
	SpO_2_ (%)	80.5882	5.77011	
Comparison of the Gold standard ABG - PaO_2_ and Pulse oximetry readings (SpO_2_)				
		Mean	Standard deviation	P-value
Pair 2	PaO_2_ (mm Hg)	61.0294	5.01178	0.043*
	SaO_2 _(%)	84.4118	4.24346	
Comparison of the Gold standard ABG - PaO_2_ and Pulse oximetry readings (SpO_2_)				
		Mean	Standard deviation	P-value
Pair 3	PaO_2_ (mm Hg)	61.0294	5.01178	<0.001***
	SpO_2 _(%)	80.5882	5.77011	
Comparison of the BP and Pulse oximetry readings (SpO_2_)				
		Mean	Standard deviation	P-value
Pair 4	BP (mm Hg)	109.6863	26.69037	<0.001***
	SpO_2_ (%)	80.5882	5.77011	
Comparison of the Gold standard ABG - SaO_2_ and BP				
		Mean	Standard deviation	P-value
Pair 5	BP (mm Hg)	109.6863	26.69037	<0.001***
	SaO_2 _(%)	84.4118	4.24346	

While comparing the mean values of SaO_2_ and SpO_2_, this difference was statistically very highly significant. While comparing the mean values of PaO_2_ and SaO_2_, this difference was statistically significant. While comparing the mean values of PaO_2_ and SpO_2_, this difference was statistically significant. While comparing the mean values of BP and SpO_2_, this difference was statistically highly significant. While comparing the mean values of BP and SaO_2_, this difference was statistically very highly significant.

Among the study population with regards to the correlation factor, there is notably a very high and strong positive correlation between SaO_2_ and SpO_2_ and between SpO_2_ and PaO_2_. There was a negative correlation between SpO_2_ and Finger abnormality and between SpO_2_ and Blood pressure (Table 4).

**Table 4:** Correlation of the values among both the procedures *Statistically significant; **Statistically highly significant; ***Statistically very highly significant

		SpO_2_
Age (in years)	Pearson Correlation	0.028
	P-value	0.777
	N	102
Hemoglobin (g/dL)	Pearson Correlation	0.306
	P-value	0.002**
	N	102
BP (mm Hg)	Pearson Correlation	-0.403
	P-value	<0.001***
	N	102
pH	Pearson Correlation	0.483
	P-value	<0.001***
	N	102
PaO_2_ (mm Hg)	Pearson Correlation	0.800
	P-value	0.043*
	N	102
PaCO_2_ (mm Hg)	Pearson Correlation	0.345
	P-value	<0.001***
	N	102
HCO_3 _(mEq)	Pearson Correlation	-0.215
	P-value	0.030*
	N	102
SaO_2 _(%)	Pearson Correlation	0.842
	P-value	<0.001***
	N	102
Finger abnormality	Pearson Correlation	-0.797
	P-value	0.008**
	N	102

Among the study population with regards to the correlation factor, there is notably a very high and strong positive correlation between SaO_2_ and SpO_2_ and between SpO_2_ and PaO_2_. There was a negative correlation between SpO_2_ and finger abnormality and between SpO_2_ and blood pressure.

Statistical significance vs clinical significance

Hence, the likelihood of obtaining a reliable result at times when there are hypoxic patients and blood pressure is significantly low or when there are confounding factors like finger abnormalities and lesions, with regards to ABG, the accuracy and sensitivity of obtaining true positive results are high compared to the finger pulse oximeter. Additionally, SpO_2_ can be used at times during emergencies but should not be relied upon as a standard procedure.

## Discussion

The gold standard for learning about a critical patient's oxygenation, ventilation, and acid-base status is an ABG analysis. ABG may be used instead of a pulse oximeter in patients with acute illnesses who have a SpO_2_ below 90%; however, this may not always be the case when people are experiencing acute issues. The study contends that a pulse oximeter is an ineffective substitute for an ABG analyzer in patients with SpO_2_ below 90% since it cannot accurately estimate SaO_2_ or assess oxygenation. One of the most crucial components of patient management is the evaluation of the patient's oxygen saturation level, which is used to determine whether to start or increase oxygen therapy. There are some drawbacks to measuring ABG analysis, including the necessity to palpate the artery, the requirement for many punctures, an increased risk of infection, and the lack of a continuous monitoring procedure. A pulse oximeter, on the other hand, can be used to continually monitor the patient's saturation level while simultaneously making immediate attempts to rectify hypoxemia. It is also patient-friendly, non-invasive, and painless. The two basic assumptions of the pulse oximeter technology are that arterial blood flow is the only source of tissue pulsations in the body on which a pulse oximeter is used, and that hemoglobin exists in two states, either as hemoglobin or oxyhemoglobin.

A normal pulse oximetry saturation is regarded as being greater than 95%, albeit this definition may change depending on the patient's comorbidities. For patients with COVID-19, oxygen therapy is frequently titrated to maintain a SpO_2_ of at least >88%. Under usual circumstances, PaO_2_ can be predicted from SpO_2_ using a conventional oxyhemoglobin dissociation curve. The ABG measurement is more precise in these circumstances, such as carbon monoxide poisoning, where the pulse oximeter reading may not adequately reflect the oxygen-carrying capacity. Some COVID-19 patients have displayed the unsettling false assurance of having a 100% SpO_2_ when their PaO_2_ is less than 50 mm Hg. To prevent avoidable mistakes, it is crucial to consider when to rely on a saturation probe and which individuals will need an arterial blood analysis.

The accuracy of comparing the oxygen saturation measured by pulse oximetry with the ABG has been the subject of numerous studies. The data made available allow for a thorough evaluation of the effectiveness and performance of the proposed technique, and a comparison with the results obtained provides a clear image of which test should be used in the event of abnormalities. In contrast to our investigation, Nessler et al. [4] observed that the frontal pulse oximeter sensor was more reliable than the pulse oximeter on the fingers in detecting SpO_2_ (n=89%) in patients receiving vasopressors. Wilson et al. [5] advocated the use of ABG rather than SpO_2_ after observing a 2.7% discrepancy between SpO_2_ and SaO_2_ in emergency patients presenting with severe sepsis and septic shock. This was consistent with the findings of our investigation, in which even we found a respectable discrepancy between the SpO_2_ and SaO_2_ levels (the mean SaO_2_ value was 84.41± 4.24). The average SpO_2_ levels were 80.58 ± 5.77).

According to Jensen et al., recent meta-analysis of 74 trials (1976 to 1994) [6] on the value of SaO_2_ revealed by pulse oximetry, pulse oximeters were accurate to within 2% of SaO_2_ in the range of 70%-100% SaO_2_. Our study confines to almost similar results with the aforementioned vast study since even our study had almost at least 10%-30% similarity between SaO_2_ and SpO_2_ values as they almost change during extreme chronic conditions and in general - hypotensive conditions only. The Carter et al. [7] research discovered that pulse oximetry performance deteriorated below a SpO_2_ of 75%, which was consistent with the findings of our study. The readings tended to decrease below a saturation point of 75%, indicating that the pulse oximeter's performance was less than optimal compared to that under typical circumstances. The current study found an almost significant difference between the SpO_2_ and SaO_2_ values, which was consistent with Chiappini et al.'s investigations [8]. They found that the pulse oximeter's accuracy was only adequate for SpO_2_ readings under 82%, which was also true in the current study. A study on COPD patients by Razi and Akbari [9] included 152 participants. A 90.8% accuracy rate, a 93.3% sensitivity rate, and an 89.1% specificity rate were all achieved via pulse oximetry. The study asserted that pulse oximetry has high accuracy in estimating SaO_2_ in pulmonary disorders with SpO_2_ at 80%, in contrast to our investigation, which discovered that accuracy is estimated when SpO_2_ is larger than 90%. The results, however, are in line with those of our study of patients with SpO_2_ 80%, which convinces us that utilizing a pulse oximeter as a substitute for an ABG analyzer to measure oxygenation is unsuitable.

In a study by Kanai et al. [10] that comprised 20,717 ABG samples from ICU patients, SpO_2_ likewise had a greater value when compared to SaO_2_. For the purpose of preventing hypoxemia, reducing mortality, and improving morbidity, the researchers advised keeping SpO_2_ above 92%. As the primary goal of the current study was to analyze the two methods separately, it was contrasted with the aforementioned study. In a similar way, several studies show that pulse oximeters are untrustworthy at low saturations because, as SaO_2_ declines, bias increases and precision (the standard deviation of the differences) decreases, increasing SpO_2_'s SaO_2_ overestimation. There are many explanations offered as to why pulse oximeter performance is so subpar at low saturations. One of the reasons is the little variations in light-emitting diode output wavelength, which at low saturations result in proportionately larger errors. Another is that measurements of transmitted red light as opposed to infrared light at low saturations yield proportionally bigger inaccuracy due to the high extinction coefficient of reduced hemoglobin.

Elhossieny [11] conducted a study to identify the factors affecting the accuracy of pulse oximetry (SpO_2_) relative to arterial oxygen saturation SaO_2_. The study included 70 patients who were admitted to the surgical and emergency ICU. The results showed that there is a reduced gap between SpO_2_ and SaO_2_ in ABGs, indicating that SpO_2_ is more accurate than SaO_2_. Age, concurrent hypoxia, mechanical ventilation, vasopressor usage, oxygen mask use, and other factors all affected accuracy; sex and sepsis, however, had no statistically significant effects.

A study by Rauniyar et al. [12] examined ABG and pulse oximetry measurements of arterial oxygen saturation in ICU patients. Out of 101 individuals, 26 (25.7%) were hypoxemic based on SpO_2_ readings. The mean SaO_2_ and SpO_2_ values were 93.22% and 92.85%, respectively. Among those with SpO_2_ below 90%, mean SaO_2_ and SpO_2_ were 91.63% and 87.42%, respectively. For SpO_2_ below 80%, mean SaO_2_ and SpO_2_ were 63.40% and 71.80%, respectively. Non-hypoxic individuals had mean SpO_2_ and SaO_2_ of 95.773% and 95.654%, respectively. The agreement rate between SpO_2_ and SaO_2_ was 83.2%, and the sensitivity and specificity of pulse oximetry were 84.6% and 83%, respectively. The study suggests that pulse oximetry is highly accurate for measuring oxygen saturation (SpO_2_ > 90%) and can replace ABG measurements.

The purpose of the 2016 study conducted by Pentakota and Amalakanti [13] aimed to assess the effectiveness of portable pulse oximetry in identifying hypoxemia in emphysema and chronic bronchitis among 50 COPD patients in an Indian hospital. The researchers compared arterial oxygen saturation (SaO_2_) measured by a blood gas analyzer with oxygen saturation (SpO_2_) assessed by a pulse oximeter. Pulse oximetry showed promise, with high sensitivity (84.60%) in identifying hypoxemia in respiratory failure. However, in the comparison between chronic bronchitis and emphysema patients, pulse oximetry had lower sensitivity (82% vs 85%) and positive predictive value (69% vs 85%) in chronic bronchitis. The study concluded that invasive ABG analysis was more accurate than pulse oximetry. It also noted the higher variability of pulse oximeter readings in chronic bronchitis patients, suggesting limitations in using pulse oximetry as a diagnostic tool for this condition.

In a 2022 study by Rose et al. [14], the accuracy of pulse oximetry (SpO_2_) in determining oxygen saturation levels (SaO_2_) was investigated in COVID-19 patients admitted to ICUs and HDUs. The study found that SpO_2_ readings alone are not entirely reliable for determining SaO_2_ levels in COVID-19 patients. The researchers observed a bias between SpO_2_ and SaO_2_ measurements, with a mean difference of 0.86%. The study concluded that ABG analysis should be used alongside pulse oximetry to accurately assess oxygen saturation levels in COVID-19 patients in ICU/HDU settings.

In the current experiment, there was a significant connection between the two testing techniques when oxygenation saturation rose above 90%. ABG may be substituted with pulse oximetry in patients with acute diseases and SpO_2_ levels below 90% due to pulse oximetry’s high SaO_2_ estimation accuracy. According to the study, a pulse oximeter cannot effectively replace an ABG analyzer in patients with SpO_2_ < 90% since it is unable to precisely quantify SaO_2_ or gauge oxygenation. Numerous research has looked at the reliability of comparing the oxygen saturation measured by pulse oximetry with ABG [15,16].

Strengths of the study

Our study has several advantages. Each patient had a single measurement taken from a pair of them. There was essentially no time difference between the two measures because they were obtained concurrently, and the ABG analysis was completed right away. Fluctuations in oxygen levels that might have occurred over time were eliminated because the measurements were collected concurrently. This helped to enhance the validity and eliminate any bias from repeatedly measuring the same subject to acquire data. SpO_2_ can overstate SaO_2_, particularly in people with dark skin when saturation is poor.

Limitations

For instance, there may have been temperature control problems as a consequence of pinched or blocked tubing inside the analyzer or as a result of blockages or gaps in the sample stream. When compared to analyzers in a core or satellite laboratory, point-of-care testing equipment may be more susceptible to analytical errors and have a greater rate of error. Small sample size. All blood gas analyzers need to be maintained regularly by the manufacturer's instructions; the schedule should be modified to fit the requirements of the specific facility. This involves regular upkeep that is carried out daily, weekly, or monthly. If there are issues with quality control or worries about the analyzer's performance, corrective maintenance is necessary. The following list includes some of the recognized errors that might occur in our investigation. In accordance with the information given above, pulse oximetry is a painless, non-invasive, cost-effective continuous monitoring technique that can be used as a good substitute for an ABG, especially when SpO_2_ is below 90%, and has an accuracy of about 83.2%. However, it is not a good substitute for determining arterial oxygen saturation in situations with low oxygen saturation (SpO_2_-90%) and a critical state and is a poor predictor of hypoxemia.

Recommendations

More study is required to determine the accuracy and effectiveness of pulse oximetry in a large sample. Furthermore, although pulse oximetry evaluates arterial oxygenation, ventilation is not assessed by it. Therefore, an ABG assay is required to confirm any clinical suspicion of alveolar hypoventilation.

## Conclusions

Thus, the study's findings show that, despite the portable, recently developed pulse oximeter's great accuracy in measuring oxygen saturation with SpO_2_ > 90%, the ABG method continues to be the Gold Standard process for reliably assessing clinical parameters under all circumstances. The study contends that, in contrast to the ABG analyzer, the precise determination of SaO_2_ and the assessment of hypoxemia by pulse oximeter is not a good option in patients with SpO_2_ < 90%. As a result, if alveolar hypoventilation is clinically suspected, pulse oximetry needs to be supported by ABG studies.
